# New insights into cerebellar dysfunction in patients with delusional disorder: A systematic review

**DOI:** 10.1192/j.eurpsy.2023.808

**Published:** 2023-07-19

**Authors:** A. González- Rodríguez, A. Guàrdia, A. Alvarez, M. Natividad, C. Pagés, C. Ghigliazza, E. Román, B. Sánchez, J. A. Monreal

**Affiliations:** 1 Mental Health, Mutua Terrassa University Hospital. University of Barcelona (UB). CIBERSAM; 2 Mental Health, Mutua Terrassa University Hospital. University of Barcelona (UB). CIBERSAM. Inst. Neurosc. UAB, Terrassa, Spain

## Abstract

**Introduction:**

The cerebellum has been implicated in cognitive, affective and motor functions, including emotion regulation, executive control and sensorimotor processing. In schizophrenia, cerebellar dysfunction has been associated with treatment resistance and clinical features. However, few studies have been focused on delusional disorder (DD).

**Objectives:**

Our main purpose was to review the evidence available on cerebellum abnormalities and dysfunctions in patients with DD.

**Methods:**

A systematic review was conducted through PubMed, Scopus and ClinicalTrials.gov (inception-June 2022) according to the Preferred Reporting Items for Systematic Reviews and Meta-analyses (PRISMA) directives. The following search terms were used: cerebellum OR cerebellar AND (“delusional disorder” AND paranoia). Reference lists from included studies were hand-checked to find other potential relevant papers.

**Results:**

Six studies were included from a total of 119 retrieved records (PubMed: 52, Scopus: 66, ClinicalTrials.gov: 1). Study 1:Patients with DD somatic type (n=14) presented a decreased gray matter volume in cerebellar lobules compared to healthy controls (HC) (n=32, left lobule VIIIa) and non-somatic DD (n=18, lobule V). Cerebellar volumes did not seem to differ between HC and non-somatic DD. Study 2:Abnormalities of voluntary saccadic eye movements, linking frontal and cerebellar functions, were found in DD patients (n=34) compared to HC (n=40). Study 3: Abnormal smooth pursuit eye movements in DD (n=15) compared with HC (n=40) and similar to schizophrenia (n=40). Case reports (n=3): DD associated with Dandy-Walker variant (partial vermian hypoplasia), unruptured intracerebral aneurysm of basilar artery, and megacisterna magna.

**Conclusions:**

Cerebellar deficits in patients with DD has been reported, particularly in those presenting somatic delusional contents.

**Disclosure of Interest:**

None Declared

